# Supplementary material for: Learning curve and surgical outcome of robotic assisted colorectal surgery with ERAS program
Source: Sci Rep. 2022 Nov 29;12:20566. doi: 10.1038/s41598-022-24665-w (PMC9709162; doi:10.1038/s41598-022-24665-w)
Supplement: Supplementary file 1 — Supplementary Information 1. [file 41598_2022_24665_MOESM1_ESM.zip › LSC Raw data-Ñ[▒K/094/O2_ñw╝╨░O▒Kñσ.pdf]

正式摘要(電子簽章：000000199577743)

W55 / W55

臺中榮民總醫院

Operation Note

[redacted]

- 手術日期：2020/09/24
- 手術醫師：劉嘯天，CR 陳映菁
- Anesthesia：GE
- Position：Supine
- Aseptic agents：Soap，Betadine Alcohol

Diagnosis

- Pre-operative diagnosis : GB Stone
- Post-operative diagnosis : Ditto.

Surgical Indication

- - Prevention from inflammation.

Operation

- - Laparoscopic Cholecystectomy

Finding

- - Pogmented stones,
  - No liver cirrhosis,
  - EBL: minimal, no transfusion.

Procedure

- Under general anesthesia, patient was put into supine position. CRS performed surgery first. I was consulted for cholecystectomy. Calot's triangle was dissected. Both cystic duct and artery were ligated. Gallbladder was resected en-bloc. CRS doctor took over for further surgery.
- Estimated blood loss: minimum
- 器械數量: 正確
- 紗布數量: 正確

Image

- 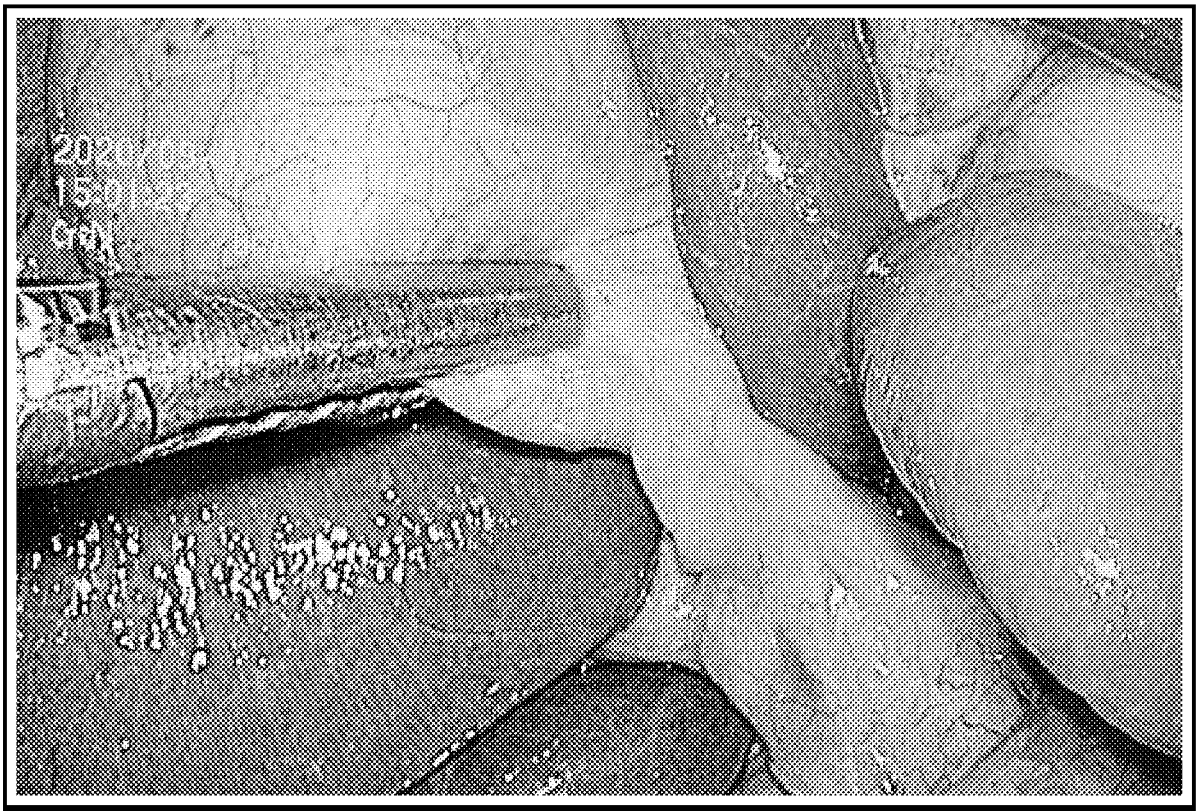

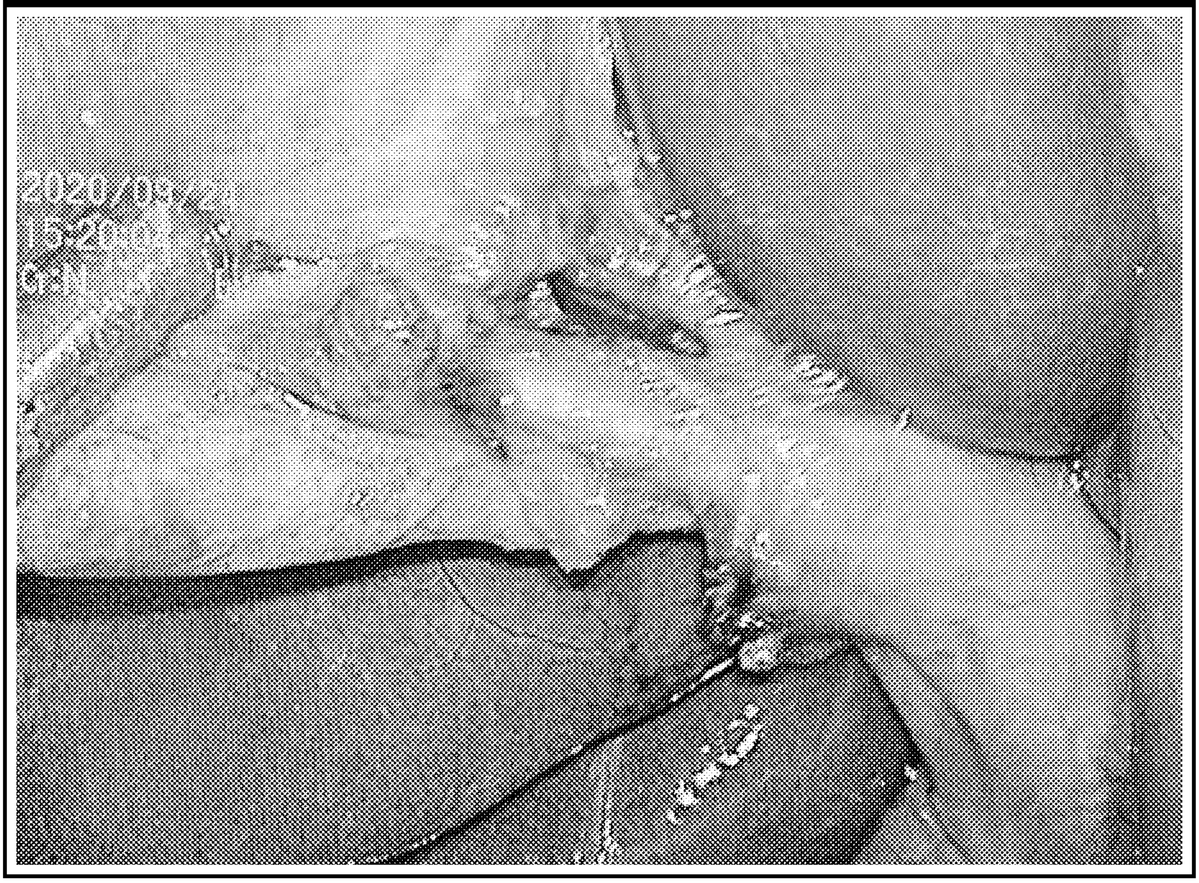

編輯主任 2062F蔣鋒帆、主治 2178L劉嘯天

正式摘要(電子簽章：000000199577743)

W55 / W55

doctorRank:B
